# Application and progress of three‐dimensional bioprinting in spinal cord injury

**DOI:** 10.1002/ibra.12005

**Published:** 2021-12-11

**Authors:** Qiu‐Qiu Xia, Hao Yuan, Ting‐Hua Wang, Liu‐Lin Xiong, Zhi‐Jun Xin

**Affiliations:** ^1^ Zunyi Medical University Zunyi Guizhou China; ^2^ Department of Orthopaedic Surgery Affiliated Hospital of Zunyi Medical University Zunyi Guizhou China; ^3^ Institute of Neuroscience and Animal Zoology Department Kunming Medical University Kunming Yunnan China; ^4^ Jinzhou Medical University Jinzhou Liaoning China; ^5^ Department of Anesthesiology, Translational Neuroscience Center, Institute of Neurological Disease, West China Hospital Sichuan University Chengdu Sichuan China; ^6^ Department of Anesthesiology Affiliated Hospital of Zunyi Medical University Zunyi Guizhou China

**Keywords:** 3D bioprinting, scaffold, spinal cord injury, three‐dimensional bioprinting

## Abstract

Spinal cord injury (SCI) is a central nervous system disorder that can lead to sensory and motor dysfunction, which can seriously increase pressure and economic burden on families and societies. The current SCI treatment is mainly to stabilize the spine, prevent secondary damage, and control inflammation. Drug treatment is limited to early, large‐scale use of steroids to reduce the effects of edema after SCI. In short, there is no direct treatment for SCI. Recent 3D bioprinting development provides a new solution for SCI treatment: a series of spinal cord bionic scaffolds are being developed to improve spinal cord function after injury. This paper reviews the pathophysiological characteristics of SCI, current treatment methods, and the progress of 3D bioprinting in SCI. Finally, its challenges and prospects in SCI treatment are summarized.

## Graphical abstract

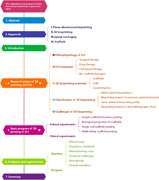

## 1 INTRODUCTION

**Figure 1:**
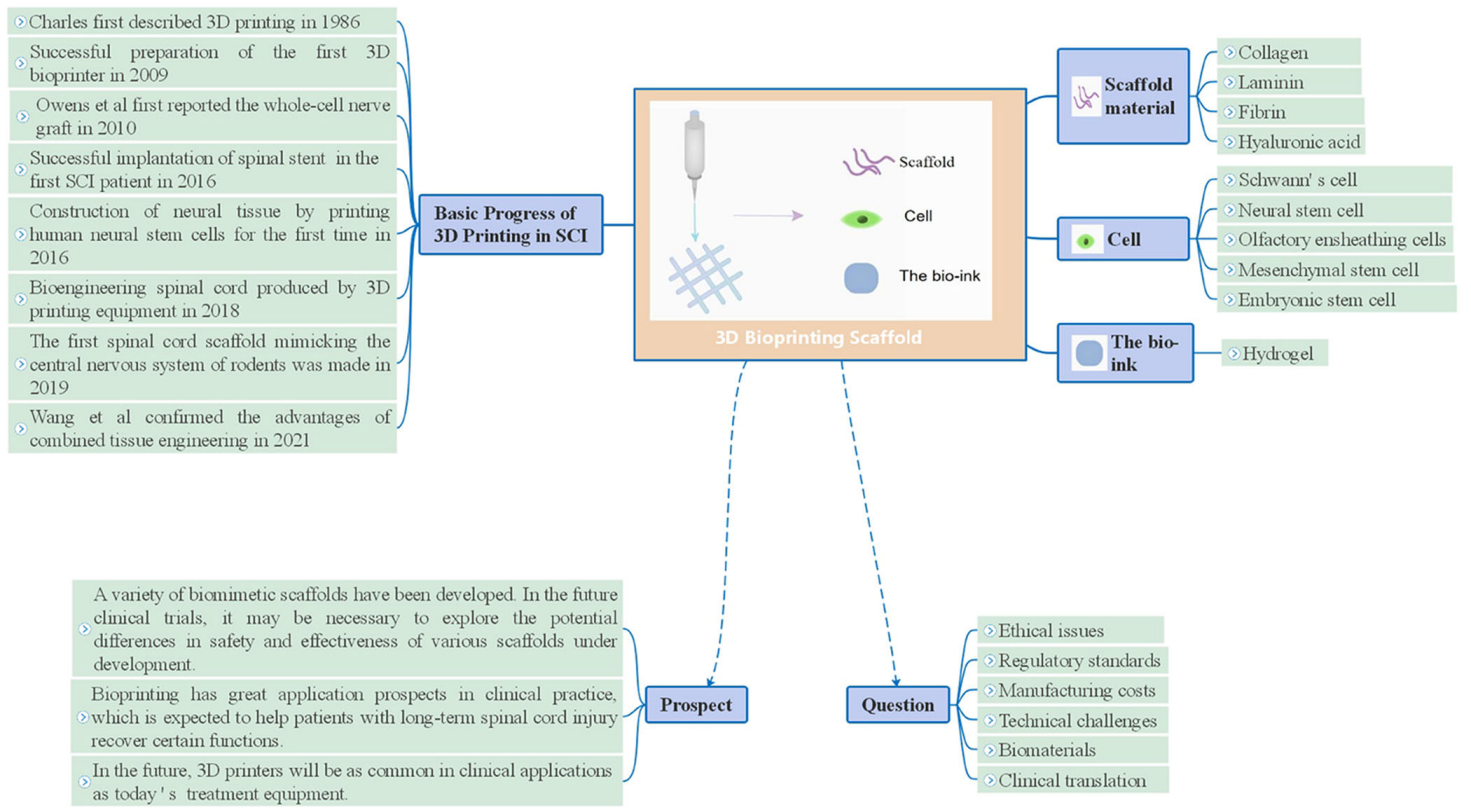
Rapid reading. SCI, spinal cord injury

Spinal cord injury (SCI) is a nervous system disorder that leads to incontinence, sensory, motor, and sexual dysfunction below the level of injury. SCI is often caused by traffic accidents, falls from heights, gunshot wounds, and knife wounds. The main complications of SCI include lung infections, urinary tract infections, and bedsores. In addition, digestive system disorders, sexual function, cardiovascular, thermoregulation disorders, and abnormal autonomic reflexes are common complications Figure 1.^1^

Globally SCI incidence continues to rise.^2^ Each year, from 250,000 up to about 500,000 people are diagnosed with SCI, indicating a 15%–40% annual incidence rate.^3^ In developed countries, one new SCI is confirmed every 16 min.^4^ In China, the incidence of SCI is increasing year by year with an annual incidence rate of 60.6 per million.^5^

In addition to varying degrees of sensory loss, decreased mobility and functional independence,^6^ there is an increased incidence of depression,^7^ less disability management and self‐efficacy,^3^ and lower quality of life.^8^ At the same time, most SCI disabilities are for life.^5^ In this scenario, the life‐long economic cost of SCI patients ranges from $1.5 million to $3 million.^9^ For instance, Harvey et al.^10^ estimated that the average hospitalization cost for each SCI patient is $95,203. Even after recovery, it will take an average of $2958 for hospital fees and $4908 for other medical expenses each year. SCI not only brings huge psychological burden to patients and their nursing staff, but also leads to high economic costs and the loss of family labor. In this scenario, SCI is the root cause of poverty for many families, thus underscoring the urgent need for effective treatment.

However, the human central nervous system cannot repair itself, and regeneration is extremely difficult. In most cases, SCI results in irreversible loss of motor and sensory functions. Due to the complex spinal structure and composition, SCI takes long time to complete a long recovery cycle. In addition, its treatment is still a problem that needs to be solved.^11^ Current surgeries, drugs, and stem cell treatments are not ideal for SCI treatment. Although a large number of studies and many regenerative therapies have shown positive results in animal models, the global medical community is yet to approve a viable option for SCI patients.

Meanwhile, in the past 10 years, 3D bioprinting technology advanced dramatically in various fields and provided new approaches for SCI repair treatment. For instance, 3D bioprinting uses rapid prototyping system to print cells, tissues, and various biological materials through layer‐by‐layer printing, microextrusion printing, stereolithography (SLA), inkjet printing, and laser‐assisted bioprinting (LAB). This technology makes it possible to create biological structures that mimic natural tissues and organs.^12^ Thus, 3D bioprinting has become a promising field for spinal cord tissue engineering.

This review summarizes the latest and ongoing research on 3D bioprinting in SCI, as well as their clinical progress, challenges, prospects, and potentials in SCI treatment. Finally, the special challenges and limitations of the transition from laboratory research to clinical applications are discussed.

## 2 RESEARCH STATUS OF 3D PRINTING AND SCI

### 2.1 Pathophysiology of SCI

The SCI pathological process includes two stages: primary injury and secondary injury. Firstly, primary injury is the direct mechanical destruction of spinal cord tissue and includes traction injury, demyelination, and necrosis of neurons and axons due to pressure. This is accompanied by central gray matter hemorrhage and immediate microvascular damage. In addition, there will be ischemia, edema, inflammatory pathway chemical release, and electrolyte transfer. Subsequently, the initial mechanical damage is compounded with necrosis and apoptosis, which damages surrounding survival neurons and further hinders the recovery of penumbra neurons as well as renders functional recovery impossible.^13^

The pathophysiological process of secondary injury aggravates the primary injury and inhibits regeneration, endogenous repair, and remyelination. This includes a variety of pathophysiological mechanisms including inflammation, local hemorrhage, ischemia, glial scar formation, lipid peroxidation, edema, axon demyelination, ion imbalance, production of free radical, necrosis, and programmed cell death.^14^

After SCI secondary injury is exacerbated by peripheral and permanent activation and recruitment of inflammatory cells. This secondary injury progresses from acute phase to subacute and chronic phases. The pathophysiological process of each phase is different.^15^ After SCI, secondary injury causes excessive accumulation and proliferation of microglia and astrocytes, thus forming solid glial scars and reducing nerve regeneration. This is one of the current hotspots in SCI treatment.^16^

## 3 SCI TREATMENT

In terms of effective treatment options, there is no clear cure for SCI at present. The current treatment plan for SCI is to stabilize the spine, prevent secondary damage, and control inflammation. The major treatment options include surgery, drugs, and stem cell therapy. Other methods include the use of growth factors and inhibition of inflammation and hypertrophic scars.^17^, ^18^

### 3.1 Surgical therapy

In terms of correcting spinal instability, evidence shows that early surgery within 24 h after SCI can stabilize the spine, prevent further injury, improve the spinal cord status,^19^ and reduce postoperative complications.^20^ There is little clinical progress in treating neurological damage to ultimately improve the prognosis of SCI patients.^21^ In addition, research indicates that surgical decompression or stabilization still lacks evidence on solid benefits for SCI patients.^22^

### 3.2 Drug therapy

Moreover, while the use of various pharmacology and cell‐based therapies have shown preclinical success, these are still in the early stages.^23^

At the same time, various drugs have been tested without clear and obvious results in improving SCI. Although many pharmacological therapies have been studied, so far, clinical trials of drugs that provided effectively for SCI are still in early‐stage testing. Administration of high‐dose steroids such as sodium succinate methylprednisolone (MPSS) is the only drug approved for acute SCI. However, the safety and effectiveness of this treatment is controversial, and studies show that it may increase gastrointestinal bleeding.^24^, ^25^

### 3.3 Cell‐based therapy

Stem cell transplantation is one of the most promising treatments for SCI. The most commonly used transplanted cells are Schwann cells (SC),^26^ progenitor cells,^27^ neural stem cells (NSC),^28^ oligodendrocyte precursor cells,^29^ bone marrow mesenchymal stem cells,^30^ and olfactory ensheathing cells.^31^ After SCI, transplanted stem cells can improve functional recovery by axon and myelin regeneration^28^ and by facilitating neuronal synaptic transmissions. Early clinical trials indicate that cell transplantation is feasible and safe.^28^ However, the safety and long‐term efficacy of this treatment mode require further verification. For instance, after cell transplantation, there are issues such as ectopic migration and fixed values,^32^ low survival rates,^33^ uncontrolled cell differentiation,^34^ and high research costs^35^ that can lead to great difficulties in clinical conversion, thus halting therapy development at the experimental stage, which means that effective treatment cannot be provided to more patients.

### 3.4 Bioscaffold therapies

Due to recent developments in material science, a variety of biological scaffolds have been developed. For instance, the main biomaterial structures currently being studied include collagen, chitosan, fibrin, poly (lactide‐co‐glycolide) (PLGA), and hydrogel.^36^ Moreover, implanting biological spinal cord scaffolds into SCI sites can mechanically stabilize spinal cord tissues, provide viable environments for host cells interactions,^37^ fill SCI voids, and guide axon regeneration.^38^ At the same time, these scaffolds can also serve as carriers that transport therapeutic substances such as cells,^39^ drugs,^40^ or growth factors^41^ to SCI sites. However, despite rapid developments in bioscaffold research in the past decade, most studies remain in the animal experiment stage.^42^ In short, there is still a long way to go before these are approved for use in clinical treatments.

## 4 3D BIOPRINTING MATERIALS

The spinal cord bionic scaffold is a new SCI treatment strategy. Traditional scaffold production cannot imitate the complex structure of natural tissues, nor can it place different types of cells in required positions on the scaffold.^43^ However, 3D bioprinting is an innovative tissue engineering technology that allows the printing of 3D scaffolds and generating microstructures that can promote and guide the growth of spinal cord axons.^44^

In addition, 3D bioprinting can print biological links (hydrogels loaded with cell‐type biomaterials) in a predesigned way as well as accurately control the composition and spatial distribution of cells and biological materials.^45^ The materials used in 3D bioprinting include scaffold materials, cells, and growth factors (see Table 1).

**Table 1:** Common biomaterials for 3D bioprinting spinal cord biomimetic scaffolds

Composition of bionic spinal cord scaffold	Constructional material
Holder	
Natural indigo material	Collagen, laminin, fibrin, hyaluronic acid, alginate, chitosan, self‐assembled polypeptides
Synthetic material	Polyglycolic acid, polylactic acid, lactic acid‐glycolic acid
Cell	
Stem cell	Muscle‐derived stem cells, embryonic stem cells, neural stem cells, mesenchymal stem cells
Nonstem cells	Schwann cells, olfactory ensheathing cells, fibroblasts, endothelial cells
Growth factor	
Nerve growth factor, BDNF, NT‐3, neurotrophic factor	

Abbreviations: BDNF, brain‐derived neurotrophic factor; NT‐3, neurotrophins‐3.

### 4.1 Scaffolds

The spinal cord bionic scaffold can provide a microenvironment for cells as well as promote axon regeneration at the injured site.^46^ In this context, researchers should consider the degradation, biocompatibility, and biomechanical properties of materials, as well as the controlled release of extracellular proteins and growth factors that may be added.^47^

Some key studies used natural polymers as scaffolds. For instance, some commonly used natural scaffolds include hyaluronic acid, chitosan, and hydrogel in addition to collagen, laminin, and fibrin, all of which exhibit biocompatibility, biodegradability, and low immunogenicity,^48^ as well as good biological activity. These natural scaffolds have the ability to work with transplanted cells and improve survival and differentiation.^47^ For example, compared with synthetic biomaterials, polylactic acid, and other artificial stents, there are usually compatibility and potential toxicity issues, but mass production and control of mechanical properties are viable.^49^ In summary, the ideal scaffold must be easy to transplant into the injured spinal cord, show good biocompatibility, low immunogenicity, and biodegradability. At the same time, a natural scaffold must also have mechanical properties that enable cell adhesion and axonal regeneration.

### 4.2 Cells

Cell culture has been used to create tissue‐like structures that allow SCI regeneration. These include nonstem and stem cells.^50^ The former include SC, olfactory ensheathing cells, fibroblasts, and endothelial cells, while the latter includes muscle‐derived stem cells, embryonic stem cells,^51^ NSCs, and mesenchymal stem cells.^52^

Cell transplantation has been safely implemented in the subacute and chronic human SCI environments.^28^ Implanting a three‐dimensional (3D) bionic scaffold containing stem cells at the injured site immediately after an injury can enhance regeneration.

### 4.3 Growth factors

In the application of 3D bioprinting scaffolds for SCI interventions and treatments, the use of nerve growth factors, brain‐derived neurotrophic factor (BDNF), neurotrophins‐3 (NT‐3), neurotrophic factors, and the like are also very important in addition to cells and scaffolds.^53^

## 5 CLASSIFICATION OF 3D BIOPRINTING

Additive manufacturing, commonly known as 3D printing, is the process of adding layers of materials to create 3D objects using computer controls. This process continues to drive major innovations in many industries.^54^ For instance, bioprinting is an approach that replaces 3D printed materials with biological materials such as living cells, proteins, and so on.

Both bioprinting and biomanufacturing methods allow the design, creation, and production of complicated 3D tissues and organs to overcome the restrictions of traditional tissue engineering methods.^55^ These technologies can be used to make scaffolds that allow cells to grow on them so that they can be implanted in the body in the future. As such, 3D bioprinting technology is a powerful tool for tissue construction in the field of tissue engineering. Since the bioprinting process requires strict environmental conditions, each step must be completed within a completely sterile environment.

3D bioprinting technology can be classified into the following four categories according to the basic working principle of manufacturing functional tissue constructors:^56^ (1) Inkjet‐based bioprinting: Most inkjet bioprinters use thermal or mechanical compression to generate and eject ink droplets on a substrate that supports or forms part of the final structure. Although thermal inkjet technology is simple and efficient, frequent nozzle clogging caused by gelation of biological links often hinders the smooth progress of the printing process.^57^ (2) Bioprinting based on pressure‐assist (extrusion): In research and business, pressure‐assisted or extrusion‐based bioprinting (EBB) is one of the most widely used method to create 3D cell‐loaded constructs.^58^ (3) LAB: This bioprinting approach is based on laser‐induced forward transfer, a process that uses laser energy to deposit high resolution cells on a target or ribbon.^59^ (4) Bioprinting based on SLA: As one of the first methods used to print living tissues, SLA bioprinting is a light‐assisted printing method.^60^ This process is a projection printing system that requires a projector to cross‐link the photocurable bio‐links side by side.^61^

## 6 CHALLENGES OF 3D BIOPRINTING

Although the latest developments in 3D bioprinting indicate a myriad of futures and possibilities such as creating implantable tissues and organs, the process also faces some challenges. For instance, there is a need for technological breakthroughs to improve the compatibility of resolution and bio‐related materials. The lack of materials must be addressed. In this scenario, there are few degradable printing materials that can promote cell attachment and proliferation, which means a very limited range of materials used.^62^ Moreover, there are uncertainties about the degree of the cell damage caused by various printing methods during cell deposition. In addition, after printing, there is a need for stable cell‐level vascularization and long‐term maintenance of cell viability and function.^54^

Although other bioprinting methods are emerging (e.g., noncontact and volumetric bioprinting), EBB is still the most widely used method among all 3D printing technologies because of its compatibility with an extensive range of materials including polymers, cell‐loaded hydrogels, acellular matrices, and aggregates.^58^

Moreover, the bionic 3D stent is an effective option for the repair of damaged nerves in the human body. In addition, 3D scaffolds can provide physical support to improve cell functions and the cell environment, thereby promoting the development of new tissues.

## 7 BASIC PROGRESS OF 3D PRINTING IN SCI

SCI and the regeneration of nervous system cells are some of the most urgent medical challenges facing the world today. The 3D neural model that mimics the natural extracellular matrix has become one of the most promising methods for reconstructing defective neural tissue. American scientist Charles Hull first described 3D printing in 1986 and developed the first commercial 3D printing machine.^63^ With the continuous developments in 3D printing technology, cell biology, computer science and material science, 3D bioprinting appeared as a form of tissue engineering in 2009 when the first 3D bioprinter was completed.

## 8 ANIMAL EXPERIMENTS

### 8.1 Simple scaffold structure printing

During initial studies, only a simple scaffold structure could be printed. Later, Wong et al. used a 3D printer to create macroscopic scaffolds with different structures (e.g., cylindrical, hollow‐tube, five‐channel, core‐open channel, and core‐free open channel), and implanted these scaffolds into a transected rat SCI model to study their effects on spinal cord regeneration. In vivo studies indicate that different scaffold structures have different effects on spinal cord regeneration.^64^ Later, Chen et al. used a 3D printer to create a more bionic spinal cord scaffold similar to the shape and size of a normal rat's spinal cord.^65^ Simple scaffold structure printing lays theoretical and technical foundations for the use of biological materials in scaffold fabrication.

### 8.2 Biological properties of scaffolds

From the results achieved by simple scaffold structure printing, scholars began to focus on the biocompatibility of printed scaffolds. For instance, Zhang et al. used 3D printing to create collagen‐heparin sulfate biomimetic spinal cord scaffolds. To observe cell adhesion and morphological changes, the scaffolds were then cocultured with rat NSCs for 7 days.^66^ In a related study, Sun et al. cultivated SCs on spinal cord scaffold surfaces prepared by PLGA, and then evaluated the effects of the PLGA scaffolds on cell proliferation and cytotoxicity.^67^ In the same year, Chen et al. planted NSCs on a 3D bioprinted collagen‐heparin sulfate scaffold, which were then implanted into the lesion site of spinal cord T10 transection in rats. About 2 months later, electrophysiological examination results indicate that collagen/heparin sulfate treatment further improved the pathological process, significantly increased the number of neurofilament‐positive cells, which unlike pure collagen stents, did not result in any adverse reactions.^68^

These experiments obtained some similar results. For instance, scaffold structures fabricated by 3D printing technology show high biocompatibility and viable biological characteristics. They can promote NSC proliferation and differentiation, as well as promote the proliferation of transplanted cells on the scaffolds. Moreover, implanting the printed scaffolds into spinal injury areas can significantly improve spinal cord recovery rates. In short, as an engineering scaffold for nerve tissue, printed scaffolds offer a myriad of research and application values.

### 8.3 Single‐cell scaffold printing

After biomaterial compatibility, stem cells and bioactive factors were confirmed. Researchers began to add living cells and biomolecules to the biomaterials used for 3D bioprinting of living cells. In early studies, for example, Li et al. used inkjet bioprinting technology to create collagen hydrogel scaffolds with rats' NSCs (C17.2) and vascular endothelial growth factor (VEGF). When morphological changes and migration ability of cells embedded in scaffolds were examined, results showed that, compared with cells in the control group, C17.2 cells in the printed collagen hydrogel scaffolds showed significant morphological changes, proliferation, and migration. Moreover, the biological printing of fibrin gels containing VEGF could promote the continuous release of growth factors in the collagen scaffolds. This printing method can be used to develop effective tissue regeneration applications.^69^

Following the work of Li et al., several scholars printed living cells using bio‐ink composed of biomaterials and NSCs. For example, Hsieh et al. injected NSCs embedded in polyurethane hydrogels into zebrafish suffering from traumatic brain injury;^70^ Liu et al. developed a new type of biocompatible ink by using functional chitosan, hyaluronic acid derivatives, and matrix glue. A scaffold loaded with NSC was fabricated by 3D bioprinting technology and implanted into SCI model rats.^71^ In addition, Gu et al. used microextrusion bioprinting technology to print neural tissue structures with human frontal cortex NSCs as printing materials, the first example of constructing neural tissue by printing human NSCs.^72^ In these studies, the aim was to investigate the therapeutic effects of printed scaffolds in animal models. Results indicate that 3D bioprinted hydrogel constructs with stem cells can maintain the viability of NSCs, promote axonal regeneration, and reduce glial scar deposition, restore a damaged central nervous system, and significantly restore the movement of SCI model rats.

In another study, Owens et al. reported the first biofabrication of a full‐cell nerve graft consisting entirely of cells and secretory substances, which they then implanted into rats with sciatic nerve injury, and found that the nerve graft partially restored motor and sensory functions in the rats.^73^

More recently, Koffler et al. published a paper in the *Journal of Nature and Medicine* for the first time that the microscale continuous projection printing (μCPP) method was used to fabricate spinal cord scaffolds that mimic the central nervous system structure of rodents. This formed a complex central nervous system structure that was used in spinal cord regeneration therapy. After the 3D‐printed scaffold loaded with NSCs was implanted into the damaged spinal cord, results showed that the damaged host axons were regenerated into the scaffold. At the same time, the implanted neural progenitor cells (NPC) extended the axons out of the scaffold and entered the host spinal cord, forming a new synaptic transmission that helped rats recover their motor function and motor evoked potential. In short, μCPP makes it possible for various biological materials to construct complex 3D scaffolds. Moreover, 2 mm scaffolds printed in 1.6 s can be created. This study shows that a 3D biomimetic scaffold is effective in regenerating a damaged central nervous system.^74^, ^75^

### 8.4 Multicellular scaffold printing

In August 2018, researchers at the University of Minnesota developed a multicellular neural tissue engineering method by using 3D printing equipment to produce a bioengineered spinal cord. They implanted iPSC‐derived NPCs and oligodendrocyte progenitor cells into printed scaffolds. Cell location was checked by dispensing printing. Results indicate that NSCs could differentiate and extend axons along the microstent channels.^76^ In a more recent study by Jianhao et al., homologous bone mesenchymal stem cells and SCs were placed in specific spaces and loaded into 3D scaffolds using cell gravity and diffusion effects to study the advantages of combined tissue engineering. Results indicate that this 3D integrated printing process can enable different types of materials and cells to simulate bionic tissues, as well as promote the recovery of motor function by strengthening tissue simulation to reconstruct myelinated axons.^77^

To sum up, multicellular printing in neural tissue engineering can simulate the tissue structure of the central nervous system, which can be used to create new bionic scaffolds, and which is more conducive to the reconstruction of axon connection and repair of damaged central nervous systems. These studies open new directions for developing treatments for nervous system disorders.

### 8.5 Clinical experiments

The preceding printed scaffolds were only used in animal experiments. However, in 2016, Theodore et al. first reported the use of scaffolds for clinical treatment by implanting a porous bioresorbable polymer scaffold a SCI sites and then monitored the patients for half a year after surgery.^78^ In another clinical study in the same year, Xiao et al. transplanted NeuroRegen scaffold into the spinal cord spaces of SCI patients after scar tissue resection, and monitored the patients for a year.^79^ The following year, Zhao et al. implanted NeuroRegen scaffolds with human umbilical cord mesenchymal stem cells into the glial scar resection site of patients with chronic complete SCI, and monitored the patients for a year.^80^ Results of these short‐term follow‐up studies were similar. Firstly, there were no surgical complications or obvious adverse reactions associated with stent implantation. Secondly, the patient showed partial improvement of autonomic nerve function, recovery of lower limb sensory evoked potential, enhanced trunk stability, and recovery of defecation sensation. These indicate that the stents are safe and feasible for the clinical treatment of SCI patients.

Following these 2 years of short‐term clinical studies on stent implantation, a long‐term clinical study from 2 to 5 years was reported. In 2021, collagen scaffolds containing mesenchymal stem cells were transplanted into acute and chronic complete SCI patients. Results indicate that most patients with acute SCI recovered gut and bladder sensation as well as autonomous walking ability. In patients with chronic SCI, the sensory level was expanded, while defecation reflex and sweating reflex were restored to some extent.^81^ Moreover, no serious adverse events related to functional stent transplantation were observed. Long‐term follow‐up results also indicated that functional stent transplantation can be a feasible treatment for patients with complete SCI. Despite the lack of large‐scale clinical conversion, the variety of scaffolds developed can help promote the clinical development and applications of 3D printing in the medical field.

At present, various single‐treatment interventions provide conditions for functional recovery post‐SCI, but effects are limited. However, compared to a single therapy, combination therapy combined with biological materials, stem cells, and biological molecules can provide better conditions to address many therapeutic obstacles and restore maximum post‐SCI functions.^82^

These 3D printing techniques can print living cells by combining biological materials, cells, and soluble molecules. As well, functional biological scaffolds combined with cells and soluble molecules can be produced. When these scaffolds are applied to local injury sites, various SCI treatments can provide advantages and benefits.

## 9 PROBLEMS AND EXPECTATIONS

### 9.1 Question

Although 3D bioprinting has been developing rapidly in recent years, 3D bioprinting involves other more complex elements compared to nonbioprinting approaches. The following limitations restrict the clinical implementation of bioprinting in SCI treatments and interventions.

## 10 ETHICAL ISSUES

The bioprinting procedure begins by digital scanning patients, after which patient data are converted to computer‐aided manufacturing. Thus, when many participants share a large number of medical information, there may be a loss of control and violation of confidentiality provisions.

The bioprinting process also raises concerns about the privacy of cell genes, the use or abuse of the technology in human reproduction, as well as ethical and legal concerns related to printing tissues and organs. However, due to the high demand for clinical treatment and the rapid developments of bioprinting technology and approaches, the implementation of appropriate regulatory standards can ensure ethical practice.

## 11 REGULATORY STANDARDS

3D bioprinting creates personalized products for the appropriate regulatory bodies to supervise equipment, biological products, and drugs. How to evaluate and approve these can be problematic. For instance, there are no manufacturing standards for 3D bioprinting technology. At the same time, there are no established standards and regulations applicable to this area.

## 12 MANUFACTURING COSTS

Commercial biological printers can cost from $5 to $350,000.^83^ Moreover, the complexity of the needed tissues or organs can require specialized and more expensive equipment.

Although cost can be a major constraint in the medical field, high costs will gradually decrease with growing market demands, and the market demand popularizes bioprinting.

## 13 TECHNICAL CHALLENGES

Currently, bioprinting is limited in focus to creating functional tissues that promote cell migration and maturation.^54^ At the same time, there is a lack of appropriate biotechnology to design biological links or to construct complex tissue structures such as those with multiple cell types or for the artificial reconstruction of complex central nervous systems.

## 14 BIOMATERIALS

Although researchers have made some dramatic advances in multimaterial printing, this process is not fully mature. Moreover, the lack of materials is still the foremost hurdle to 3D printing.

Due to the complexity of the central nervous system, accessing functional biomaterials suitable for central nervous system injury remains a daunting challenge.

## 15 CLINICAL TRANSLATION

So far, 3D printing has proven to be a boon in medicine such as cell‐free 3D printing technology used in plastic surgery, maxillofacial surgery, and dentistry. Moreover, clinical trials have been established to evaluate the safety and effectiveness of manufacturing devices.^84^ However, most medical fields are yet to undergo clinical transformation. This is not only due to the mentioned difficulties but also due to the dearth of scientific evidence.^85^

The onset of each new technology includes a range of challenges. To solve complex problems, it is necessary to integrate technologies in engineering, biomaterial science, cell biology, physics, and medicine. At the same time, it is believed that, after finding viable solutions, 3D bioprinting will develop more rapidly and bring great benefits to human organ transplantation and the treatment of central nervous system disorders.

## 16 PROSPECT

Bioprinting of nerve tissue has great clinical prospects for repairing SCI. However, this technology is still in its infancy. Research and clinical trials remain limited and clinical applications are rare. In this context, further studies are needed to achieve the standard and widespread clinical implementation.

On the other hand, although the field of 3D bioprinting is at an early stage, it has been successfully used for the transplantation of some tissues including multilayer skin, vascular grafts, heart tissue, bone, tracheal splints, and cartilage structures.^54^

At the same time, new generations of 3D biological printing technology such as 3D integrated printing may be conducive to functional recovery in SCI scenarios. Future clinical trials could explore the potential differences in the safety and effectiveness of various scaffolds being developed. One day, 3D bioprinting can help patients with long‐term SCI recover some functions.

Moreover, it is believed that printing spinal bionic scaffolds is only a transition stage in 3D bioprinting for SCI intervention and treatment. When 3D bioprinting breaks through the critical point of scaffold printing, it will probably be able to print nerve tissue. Then, applications of 3D bioprinting may regenerate nerve tissue and completely change the medical industry. It can be predicted that, in the future, 3D printers will be as common in clinical applications as today's standard medical equipment. However, further research in this area is still needed.

## 17 SUMMARY

The ultimate goal of SCI treatment is to maximize nerve recovery or minimize inconvenience to patients.^22^ These are challenging goals for researchers, patients, and medical staff.

As a feasible choice to improve the lives of SCI patients, 3D bioprinting technology is widely recognized by doctors and researchers around the world. However, despite significant progress in 3D bioprinting, SCI research is still relatively undeveloped. The complexity of the CNS structure means that a viable 3D printing procedure is yet to be completed.

The long‐range vision of 3D bioprinting technology is to repair nerve tissue, which is one of the most promising technical methods being explored. Due to the great excitement brought about by this development, new frontiers in the integration of neuroscience and tissue regeneration engineering can be expected to bring about a promising future to SCI patients, their doctors, caregivers, families, and friends.

## CONFLICT OF INTERESTS

The authors declare that there are no conflict of interests.

## ETHICS STATEMENT

Ethics statement is not applicable for this article.

## AUTHOR CONTRIBUTIONS

Qiu‐Qiu Xia and Hao Yuan wrote the initial draft of the paper. Ting‐Hua Wang contributed the central idea; Liu‐Lin Xiong mentioned the idea. Zhi‐Jun Xin revised the paper.

## Data Availability

Data sharing not applicable to this article as no datasets were generated or analysed during the current study.
